# When income meets faith: the development and application of the Chinese generation Z unconventional religious orientation scale

**DOI:** 10.1186/s40359-024-01835-1

**Published:** 2024-06-04

**Authors:** Wang Ziang, Jiang Jindong, Cao Xuan, Luo Yinglin

**Affiliations:** 1 Hangzhou Normal University, Zhejiang, Hangzhou, China; 2 Hong Kong Ruyi culture limited, Hong Kong, China

**Keywords:** Generation Z in China, Unconventional religious orientations, Income satisfaction, Psychometrics, Social psychology

## Abstract

This study seeks to analyze the psychological construction of Unconventional Religious Orientations and their association with individual income level satisfaction within Generation Z. Generation Z, individuals born between 1995 and 2010, grew up in a socio-cultural context marked by digitization and globalization. This study identifies three key dimensions of Unconventional Religious Orientations: religious spiritual dependence, religious instrumental tendencies, and religious uniqueness identity. By combining rootedness theory, semi-structured interviews, and literature review, we constructed and refined a set of relevant scales. Using exploratory and validation factor analyses (EFA and CFA), we verified the structural validity of the scale. The results of the analyses revealed significant negative correlations between satisfaction with income level and all dimensions of Unconventional Religious Orientation for Generation Z, suggesting that Unconventional Religious Orientation tends to diminish as income satisfaction increases. In addition, the significant positive correlations between these dimensions of religious inclination imply that they may share certain underlying factors in their psychological structure. This study not only successfully developed a set of psychometric instruments for Unconventional Religious Orientations, but also provided a new psychological perspective for understanding the dynamic interaction between economic satisfaction and religious psychological attitudes in Generation Z.

## Introduction

In this study, we focus on exploring and defining the concept of Unconventional Religious Orientations in depth, with special attention to the specific group of Generation Z [1, 2]. Generation Z, usually referred to as the younger generation born between 1995 and 2010, has grown up in a unique socio-cultural environment characterized by digitalization and globalization. The values, lifestyles, and religious attitudes of this generation differ significantly from those of their predecessors, as they place greater emphasis on pluralism, inclusiveness, and social justice, tend to question traditional authority and seek individualized lifestyles and paths of faith [1]. As digital natives, Generation Z in Chinahas an in-depth understanding of social media and network technology and is accustomed to obtaining information and communicating with others through the Internet. Their religious attitudes show greater mobility and individualistic tendencies, and they may be more inclined to explore different religious beliefs or hold non-traditional, personalized religious views [3].

In exploring this phenomenon, it is important not to lose sight of the influence of the particular cultural context of China. In China, atheism is promoted by the government and has had a profound impact on social culture and personal beliefs, although this does not mean that all people have given up their religious beliefs. In fact, many Chinese people still adhere to various religious rituals and beliefs in private. Confucianism, as a non-religious philosophical and ethical system that emphasizes family values, social harmony, and personal moral cultivation, has had a profound influence on Chinese society. In addition, Taoism and Buddhism, as systems of a more religious nature, have also had a wide cultural and philosophical impact on Chinese society. At the same time, folk beliefs are extremely rich, including the worship of ancestors, the worship of native deities such as the Land Goddess and Mazu, and activities such as feng shui and fortune-telling. These beliefs and practices are often closely linked to traditional religions and are an important part of community culture and family traditions. In modern society, although many traditional religious practices are often regarded as superstitious, this categorization may be oversimplified from an academic perspective. In fact, these practices considered as superstitions have important symbolic meanings and social functions in many cultures, demonstrating their role in maintaining social structures and cultural identities.

Relying on the previous rooted theoretical research, we found that the concept of Unconventional Religious Orientation has not yet been clearly defined in the academic field. Through systematic data collection and analysis, we identified that Unconventional Religious Orientations can be divided into three main dimensions: religious spiritual dependence, religious instrumental tendency, and religious uniqueness identity. These dimensions not only reflect the diversity of Unconventional Religious Orientations in actual behaviors and belief manifestations, but also provide us with a way to quantify and refine such tendencies.

From a psychological perspective, Unconventional Religious Orientations are seen as important factors that have a profound impact on an individual’s mental health, social behavior, and decision-making processes [1]. Therefore, the development of a precise scale to quantify Unconventional Religious Orientations for Generation Z in Chinais particularly necessary. This will not only help to fill the theoretical gap, but also facilitate our deeper understanding and application of this phenomenon. The development of the scale will integrate multiple dimensions such as religious spiritual dependence, religious instrumental tendency, and religious uniqueness identity to comprehensively capture the complexity of Unconventional Religious Orientations.

Specifically, we hypothesize that there may be a correlation between Generation Z’s Unconventional Religious Orientations and income satisfaction [3–5]. Considering the unique values and lifestyles of Generation Z, this correlation may manifest itself in their career choices, work attitudes, and economic expectations. By collecting data and applying statistical methods for exploratory and validation analyses, we aim to establish the nature and strength of the relationship between Unconventional Religious Orientations and income satisfaction.

The purpose of this study was not only to develop and validate a scale on Unconventional Religious Orientation, but also to explore the potential link between this inclination and economic satisfaction in Generation Z. The study was conducted to examine the relationship between religious inclination and economic satisfaction in Generation Z. The results of this study are summarized in the following table. This exploration is important for understanding how religiosity affects economic perceptions and career development at the individual level, but also provides insight for social psychologists and policymakers to help them understand the role of religious inclination in economic behavior and social satisfaction, especially for the younger generation. Thus, this study is not only valuable for academic research in psychology and sociology, but also provides practical guidance for understanding and responding to relevant issues in modern society.

## Methodology

### Scale item design

In this study, we utilized Grasser and Strauss’s grounded theory approach in the process of acquiring the question items of the Chinese Unconventional Religious Orientation Scale [6, 7]. This approach is applicable to exploring complex social phenomena, particularly Unconventional Religious Orientations among those who subscribe to the teachings of Taoism and Buddhism. We extracted key concepts from semi-structured interviews and document analysis through open coding, followed by spindle coding to gain a deeper understanding of the relationships between these concepts, and finally selective coding to construct a comprehensive theoretical framework [8–11].

Open coding is a fundamental step in qualitative research that requires the researcher to read and scrutinize the interview transcripts in order to identify key themes and patterns from them for initial categorization of the data. This is followed by the spindle coding stage, where the researcher integrates and relates these initial codes into broader categories or concepts, exploring causal relationships, conditions, contexts, and outcomes, such as categorizing the codes into thematic categories such as “belief avoidance” or “belief dependence”. categories. The final stage of selective coding involves aggregating the categories from the master codes into a central theme or ‘core category’, which is used to build a unified theory around the core concepts, making the findings and theoretical framework more coherent and structured. This whole process helps the researcher to gain a deeper understanding of the data and develop valid theoretical explanations.

To ensure representativeness and diversity in the study, 29 participants of different genders, ages, occupations, and religious backgrounds were included (see Table 1). Each interview lasted between 30 and 50 min and was conducted through both offline and online platforms to optimize data quality.

The study utilized a dual-mode interview methodology that included both offline and online formats. Offline interviews were conducted in quiet, minimally intrusive environments, using a KDDI RS502 recorder for accurate data collection. These environments were deliberately chosen to create an atmosphere conducive to open and unrestricted dialog. The online interviews, on the other hand, were conducted through the Tencent conferencing software. Preparatory measures ensured optimal functionality of all technical equipment for smooth communication. The interviews began with an initial consultation phase in which the researcher introduced the research topic and clarified the purpose of the interview material, aiming to create an appropriate atmosphere for discussion.

To ensure representativeness and diversity in the study, we included 29 participants with different gender, age, occupational and religious backgrounds (see Table 1). Each interview lasted between 30 and 50 min and was conducted both offline and through an online platform to optimize data quality. Throughout the study, we strictly adhered to the principles of privacy and confidentiality, ensuring voluntary participation and demonstrating a commitment to ethical research practices [12].

**Table 1:** Basic Information of Participants

Participant No.	Genders	Careers	Religious Background	Interview Length/min
P1	male	SOE employeesr	Taoism	38
P2	male	Freelance	Taoism	32
P3	female	Freelance	Taoism	29
P4	female	Schoolchildren	Buddhist	31
P5	female	Yoga Sound Therapy Teacher	Buddhist	62
P6	female	Schoolchildren	Taoism	68
P7	female	Attorney	not have	19
P8	female	Attorney	not have	22
P9	female	Attorney	not have	18
P10	female	Attorney	not have	22
P11	female	Attorney	not have	19
P12	female	Schoolchildren	not have	33
P13	female	Schoolchildren	Buddhist	47
P14	female	Schoolchildren	Buddhist	58
P15	female	Anchor (TV)	Buddhist	23
P16	male	Schoolchildren	Taoism	63
P17	female	Schoolchildren	Buddhist	42
P18	male	Attorney	Buddhist	53
P19	female	staff member	Taoism	28
P20	female	Schoolchildren	Buddhist	32
P21	female	Schoolchildren	Taoism	27
P22	male	Engineer	Buddhist	18
P23	male	Schoolchildren	Taoism	19
P24	male	Schoolchildren	Buddhist	28
P25	female	SOE employees	not have	21
P27	female	Chinese Medicine Doctor	Taoism	49
P28	male	Entrepreneur	Taoism	53
P29	female	Venture Partner	Taoism	63

Through this detailed and systematic approach, the researcher not only provided theoretical insight into the multidimensional nature of Unconventional Religious Orientation, but also succeeded in extracting key concepts from the empirical data, laying a solid foundation for the initial question production of the scale. This process exemplifies the ongoing commitment to deep understanding, accurate measurement, and ethical standards in psychological research.

Based on the model of Unconventional Religious Orientation developed by the Grounded theory and a search of the literature [13], we obtained an initial 34 conceptual entries on Unconventional Religious Orientation. After expert panel evaluation and refinement of similar concepts, the existing 20 entries were retained, as shown in Table 2.

**Table 2:** Pool of Items

serial number	conceptual	subject headings
1	conform slavishly	I am influenced by people around me in matters related to religion.
2	obsessed	I will pay money to find a guru on social media on the internet to read marriage and career, if the result is not satisfactory, I will think that this guru is not good enough and will consider changing another guru.
3	drag	I used to ask my friends or family members to go to a certain temple at a certain point in time to pray for blessings
4	extravagance and waste	I think spending more than you can afford on a pilgrimage to the Holy Land is an understandable behavior
5	lit. blind rendering (idiom); deliberate exaggeration	I’d like to recommend the religious guru I just met to my friends and spread the word in my circle of friends
6	profit seeking propensity	If I had learned to tell fortunes or predictions, I would have made money by this method
7	reparation	Going to a temple or a Taoist temple makes me feel bad if I don’t pay for it
8	flaunt	I like to mention the temples and Taoist temples I’ve visited at social events and introduce them to the masters I’ve known
9	investment climate	I think one’s luck gets better as one goes to the temple more often or spends more time and money on religion
10	flight from reality	As long as real life is stressful, I want to go to the temple and stay there.
11	shirk	“Between going to work and getting ahead, I’ll take the incense.” That’s a good reflection of where I’m at right now.
12	see, hear and obey (idiom); to take advice	I feel that if I don’t act on what the master says, everything will go wrong.
13	dogmatic and rigid	Before you do anything, you’ll always get your fortune read or check your luck.
14	passive response	In times of trouble, I find praying for divine blessings to be an effective approach
15	Authority relies on	I would like to meet a few more monks and Taoist priests in real life so that my future is very secure
16	double standard	I feel uncomfortable when people comment on my favorite religion, but I have no such qualms when commenting on other religions.
17	social isolation	I can only find a sense of belonging in the Taoist/Buddhist community
18	emotional dependence	I feel that life is painful and only by relying on the power of the gods can I feel better in my heart
19	seduce	I’ll convince everyone around me to go to some temple with me to pray for blessings.
20	paranoia	I feel like doing something related to religion at a certain time must be prioritized above all else

### Exploratory factor analysis

In the present study, we aimed to gain insight into the underlying structure of Unconventional Religious Orientations through exploratory factor analysis (EFA) [14, 15]. To this end, we crafted a questionnaire containing 20 entries that focused on measuring individuals’ attitudes toward religious practices and beliefs (see Table 3 for details). The first part of the questionnaire collected demographic information, including gender, age, and educational background. Through online platforms and social networks, we distributed 372 questionnaires to 3,143 residents living in eastern China and successfully collected 214 of them, achieving a 57% return rate. Our sample consisted mainly of males (66.4%), with a concentration of ages between 18 and 30 years old (90.7% of the total sample), and the majority of respondents had a bachelor’s degree or a specialized degree (67.8% in total).

In terms of methodology, we adopted the minimum residual method as the factor extraction method, which is suitable for revealing the underlying structure in the data and effectively handling non-normally distributed data. In addition, in order to obtain a clearer and more explanatory factor structure, we used oblique rotation methods such as the oblique maximum variance method [16, 17]. This method allows for some correlation between factors and is more suitable for revealing the interactions between complex mental constructs.

We also validated the plausibility of the factor interpretations to ensure that each factor had a clear and consistent psychological meaning. To ensure each factor from our exploratory factor analysis had a clear and consistent psychological meaning, we implemented a comprehensive validation process. Initially, using the minimum residual method and oblique rotation, we identified significant factors through eigenvalues and the scree test, effectively distinguishing substantial constructs from random variance. We then scrutinized the loadings of questionnaire items, retaining those with loadings of 0.40 or higher to ensure robust relationships with their respective factors. Subject matter experts were engaged to review the item groupings for each factor, confirming that they accurately represented the intended psychological constructs, thus ensuring content validity. Additionally, we enhanced construct validity by correlating the factors with external measures related to Unconventional Religious Orientations, such as religiosity and personal autonomy. Consistency checks were performed using Cronbach’s alpha, with factors scoring above 0.70 deemed reliable. Factors were named based on their psychological significance as determined by quantitative analysis and expert reviews; for example, a factor related to the use of religious rituals in stressful times was named ‘Religious Coping Orientation.’ Through these meticulous steps, we successfully revealed the underlying structure of Unconventional Religious Orientations using exploratory factor analysis. Although limited by sample size and diversity, the insights gained offer valuable information for understanding religious predispositions among specific groups and lay a solid foundation for future research aimed at a broader and more diverse population, enhancing our comprehension of the complexity and diversity of Unconventional Religious Orientations.

**Table 3:** Questionnaire Items

Title number	Questionnaire entry
T1.	Make a wish at a nettlesome temple or Taoist temple will be more effective.
T2.	As long as real life is stressful, you want to go to a temple or a Taoist temple and stay there
T3.	“Between going to work and getting ahead, I’ll take the incense.” That’s a good reflection of where I’m at right now.
T4.	Praying for divine blessings in times of trouble is an effective method of
T5.	I feel that life is painful and only by relying on the power of the gods can I feel better in my heart
T6.	I’ll convince everyone around me to go to some temple with me to pray for blessings.
T7.	I used to ask my friends or family members to go to a certain temple at a certain point in time to pray for blessings
T8.	Going to a temple or a Taoist temple makes me feel bad if I don’t pay for it
T9.	I’ll pay a guru to read my marriage and career, and if the result is not satisfactory, I’ll think that the guru is not good enough and will consider changing to another guru
T10.	I like to mention the temples and Taoist temples I’ve visited at social events and introduce them to the masters I’ve known
T11.	I’d like to recommend the religious guru I just met to my friends and spread the word in my circle of friends
T12.	I would like to meet a few more monks and Taoist priests in real life so that my future is very secure
T13.	I feel that if I don’t act on what the master says, everything will go wrong.
T14.	If I learn to tell fortunes or make predictions, this will definitely be one of the ways I can make money
T15.	I feel that the amount of money spent on religious matters is directly proportional to one’s fortunes improving
T16.	I think it takes a lot of religion to get what you want.
T17.	I feel that doing something related to religion at some point in time is prioritized above all else
T18.	Before making any decisions, I would take some metaphysical factors into account
T19.	Feel uncomfortable when people comment on my favorite religion, but have no such qualms when commenting on other religions
T20.	I can only find a sense of belonging in my favorite religious community

### Confirmatory factor analysis

In this study, we utilized a validated factor analysis (CFA) to assess the construct validity of the Generation Z in ChinaUnconventional Religious Orientation and Income Satisfaction Scale [18]. To ensure extensive and convenient data collection, we distributed 3,143 questionnaires through online questionnaires and social networking platforms, and took several measures to ensure the integrity and authenticity of the data, such as setting up logical consistency checking and time-stamp tracking.

Among the collected data, we conducted a rigorous data cleansing exercise to remove incomplete or inconsistent responses, and excluded responses with abnormal speed based on the time taken to complete the questionnaire. To further ensure data quality, we compared participants’ IP addresses and response patterns to rule out duplicates and mechanical responses, and verified the quality of responses by checking random samples. This series of quality control measures eventually helped us establish 2,025 valid questionnaires, with an effective return rate of 64.4%.

The geographical distribution of the sample covered three major provinces in eastern China: Anhui, Zhejiang and Jiangsu, in which the gender ratio of men and women was close to balanced, the age group was mainly concentrated between 18 and 30 years old, and the educational backgrounds were mainly undergraduate degrees with a variety of occupational backgrounds. This multidimensional sample structure not only ensures the representativeness of the study, but also provides a solid foundation for data analysis and interpretation of results.

In the model building and testing phase, we used jamovi 2.4.11 statistical software, an intuitive and powerful tool to construct and evaluate the hypothesized factor structure model. We assessed the fit of the model by calculating fit metrics such as χ²/*df*, RMSEA, CFI and TLI [19, 20]. To verify the stability and replicability of the results, we also performed cross-validation and compared them with the results of exploratory factor analysis (EFA) to confirm the consistency and accuracy of the factor structure.

Taking all these steps together, we are confident that the CFA results of this study are reliable and provide solid statistical support for understanding the relationship between Generation Z’s propensity for irrational religiosity and income satisfaction.

### A study of the relationship between income level satisfaction and unconventional religious orientation

In this study, we delved into the relationship between income level satisfaction and Unconventional Religious Orientations of Generation Z in Chinain eastern China. Through online questionnaires and social networks, we distributed questionnaires to 3,143 residents and successfully collected 2,025 copies, achieving a high recovery rate of 64.4%. These samples covered the provinces of Anhui, Zhejiang and Jiangsu with good geographical representation. The gender ratio was close to balanced and involved a wide range of occupational backgrounds, including students, civil servants, general employees to freelancers, and this multidimensional sample structure provided a solid empirical foundation for our study.

In order to accurately measure Generation Z’s Unconventional Religious Orientation and income level satisfaction, we developed a set of self-assessment scales and used the income level satisfaction scale validated by Tang et al. [21]. After Chinese cultural revisions and pre-testing, these instruments showed good applicability in the Chinese cultural context, laying a solid foundation for quantitative analysis.

In terms of data collection and processing, we conducted a comprehensive assessment of the three sub-dimensions of Unconventional Religious Orientation and income satisfaction, and meticulously reviewed and cleaned the collected data to exclude incomplete or abnormal data to ensure the accuracy of the analysis results. Next, we analyzed the correlation between the sub-dimensions of Unconventional Religious Orientation and income satisfaction by using Pearson’s correlation coefficient on the basis of descriptive statistical analysis [22, 23], and assessed the predictive role of income level satisfaction on Unconventional Religious Orientation through linear regression analysis, which revealed the interaction between the two [24, 25].

## Results

### Exploratory factor analysis

In this study, the results of exploratory factor analysis (EFA) revealed three main factors that significantly reflected the multidimensionality and complexity of Unconventional Religious Orientations. Specifically, the first factor explained 29.6% of the total variance, the second contributed 19.0%, while the third explained 15.7%, cumulatively explaining 64.2% of the total variance (see Tables 4 and 5 for details). These results exceed the minimum standard of variance explained (5–10%) typically expected for factors in psychological research, suggesting that these factors are critical in understanding the structure of the data. Further, the correlations between the three factors reveal some degree of interaction between them, which contributes to a deeper understanding of how individuals perform on different dimensions of religious orientation. In terms of model fit, the root mean square error of approximation (RMSEA) was 0.0397, which is significantly lower than the standard threshold of 0.05 [26, 27], and the TLI (Tucker-Lewis Index) index was 0.978, which is close to 1 [28, 29], both of which imply a high degree of fit between the model and the data (for details, see Table 6). In addition, the applicability of the factor analysis is supported by the results of the fitness tests, where the Bartlett’s test of sphericity has a χ² value of 2,744 with a *p*-value of less than 0.001, and the Kaiser-Meyer-Olkin (KMO) test of suitability for sampling has a result of 0.901, which is much higher than the benchmark value of 0.5 [30, 31], suggesting that there is sufficient covariance among the variables in the sample. Taken together, these indicators show that the proposed factor structure is statistically sound and valid, providing a solid foundation for understanding Unconventional Religious Orientations.

**Table 4:** EFA Factor Loading Results

Title number	F1	F2	F3	Unique residual value
T1.			0.989	0.021
T2.		0.908		0.199
T3.		0.830		0.258
T4.		0.856		0.270
T5.		0.867		0.246
T6.		0.865		0.251
T7.			0.585	0.644
T8.			0.696	0.516
T9.	0.830			0.299
T10	0.832			0.273
T11			0.604	0.637
T12.				0.770
T13.			0.569	0.679
T14.	0.838			0.251
T15.	0.811			0.282
T16.	0.880			0.256
T17.	0.889			0.234
T18.	0.870			0.263
T19.			0.623	0.603
T20.	0.896			0.203

**Table 5:** Factor Analysis

(math.) factor	SS Load	Variance %	Cumulative %
1	5.91	29.6	29.6
2	3.8	19	48.5
3	3.14	15.7	64.2

**Table 6:** Evaluation of Model Fitting

	90% confidence intervals for the root mean square error of approximation				model checking		
RMSEA	Lower limit	Upper limit	TLI index	BIC	χ²	*df*	*p*
0.039	0.019	0.056	0.978	-521	172	133	0.013

### Confirmatory factor analysis

The present study aimed to validate the structural validity of the Unconventional Religious Orientations Scale by performing a confirmatory factor analysis (CFA). The results of the analysis revealed the presence of three main factors: religious spiritual dependence, religious instrumental tendencies, and religious uniqueness identity. These factors reflect the theoretical underpinnings of the scale design and comprehensively capture the multidimensional nature of Unconventional Religious Orientations.

In the religious-spiritual dependence dimension, entries such as “As long as real life is stressful, I want to go to the temple or Taoist temple to stay there” (loading 1.58, Z-value 39.2, *p* < .001) and “Praying for the blessing of the gods is an effective method when encountering difficulties” (loadings 2.17, Z-value 46.2, *p* < .001), among others, demonstrated high loadings, suggesting that these entries were highly correlated with the factor Religious Spiritual Dependence. In the dimension of Religious Instrumental Tendency, for example, the high loadings for “I will pay a guru for marriage and career” (loading 2.26, Z value 49.5, *p* < .001) reinforced the conceptual clarity of the factor. Similarly, the results for “Going to an online temple or Taoist temple to make a wish will be more spiritual” (loading 1.67, Z-value 41.5, *p* < .001) in the Religion Unique Attribute Identity dimension similarly supported the validity of the scale. All entries had *p*-values less than 0.001, indicating that the factor loadings were statistically significant (see Table 7 for details).

In addition, analyses of covariance between factors, such as the covariance between Religious Spiritual Dependence and Religious Instrumental Tendency, was 0.589 (*p* < .001), revealing the interrelatedness of these constructs (see Table 8 for details). This finding suggests the importance of the interactions between the dimensions and their understanding of Unconventional Religious Orientations in practical applications and theoretical explanations.

For the assessment of model fit, the TLI value in this study was 0.976, the CFI was 0.979, and the SRMR was 0.034, and the RMSEA was 0.042 (90% confidence interval 0.039–0.045), which point to a good fit of the model and show that the scale is statistically robust [32, 33]. The chi-square test for the exact fit test showed a χ² = 680, with a degree of freedom (*df*) of 149 and a *p*-value of less than 0.001 [34, 35].This result suggests that there is a statistically significant difference between the theoretical model and the observed data. However, this significance must be interpreted with caution in the context of a larger sample size (2025 for this study).

Taken together, the Chinese Unconventional Religious Orientation Scale constructed in this study demonstrated good validity and reliability in terms of theoretical constructs and empirical tests.

**Table 7:** CFA Factor Loadings

factor analysis	entry (in a dictionary, encyclopedia etc.)	ratio	standard error	Z	*p*
F1. Religious Spiritual Dependence	T2.	1.58	0.040	39.2	< 0.001
	T3.	1.68	0.041	40.9	< 0.001
	T4.	2.17	0.047	46.2	< 0.001
	T5.	1.65	0.041	40.5	< 0.001
	T6.	1.62	0.041	39.5	< 0.001
F2. Instrumental tendencies of religion	T9.	2.26	0.046	49.5	< 0.001
	T10.	1.54	0.040	39	< 0.001
	T14.	1.67	0.041	40.5	< 0.001
	T15.	1.56	0.039	39.5	< 0.001
	T16.	1.6	0.040	39.9	< 0.001
	T17.	1.6	0.040	40	< 0.001
	T18.	1.61	0.040	39.8	< 0.001
	T20.	1.67	0.041	40.4	< 0.001
F3. Religious Uniqueness Identity	T1.	1.67	0.040	41.5	< 0.001
	T7.	1.57	0.040	39.4	< 0.001
	T8.	1.73	0.041	42.3	< 0.001
	T11.	1.67	0.041	40.9	< 0.001
	T13.	2.16	0.046	46.7	< 0.001
	T19.	1.7	0.041	41.9	< 0.001

**Table 8:** Factor Covariance

(math.) factor		ratio	standard error	Z	*p*
Religious spirituality	Religious spirituality	1			
	Instrumental tendencies of religion	0.589	0.017	35	< 0.001
	Religious identity	0.624	0.016	38.6	< 0.001
Instrumental tendencies of religion	Instrumental tendencies of religion	1			
	Religious identity	0.614	0.016	38.7	< 0.001
Religious identity	Religious identity	1			

^a^ Fixed factor

### The relationship between income level satisfaction and unconventional religious orientation

In this study, we delve into the relationship between income level satisfaction and Unconventional Religious Orientation, with a special focus on three dimensions of Unconventional Religious Orientation: religious spiritual dependence, religious instrumental inclination, and religious uniqueness identity. By analyzing the sample of Generation Z, we find that income level satisfaction shows a significant negative correlation with each dimension of Unconventional Religious Orientation (see Table 9 for details). Specifically, there was statistically significant support for a decrease in the propensity for religious spiritual dependence, religious instrumental tendency, and religious uniqueness identity as income level satisfaction increased.

Moreover, significant positive correlations were observed for the relationships between these three dimensions of Unconventional Religious Orientations. This suggests that psychologically structured, these dimensions may share certain underlying factors. Most significant was the correlation between Religious Spiritual Dependence and Religious Instrumental Tendency, followed closely by Religious Spiritual Dependence and Religious Uniqueness Identity and Religious Instrumental Tendency and Religious Uniqueness Identity .

Further quantitative analyses revealed a linear relationship between income satisfaction and Unconventional Religious Orientations (see Table 10 for details). Using the linear regression model, we found a compound correlation coefficient R of 0.463 and a coefficient of determination R² of 0.214, which implies that income satisfaction explains 21.4% of the variance in Unconventional Religious Orientations. In this model, the negative correlation coefficient of income satisfaction further emphasizes the significant downward trend of Unconventional Religious Orientations when income level satisfaction increases [25, 36].

Taken together, these findings provide new perspectives for understanding the complex relationship between income level satisfaction and Unconventional Religious Orientations. They not only have important theoretical implications in the fields of psychology and social sciences, but also provide rich insights for future research, especially in considering the impact of economic factors on religious attitudes and behaviors.

**Table 9:** Correlation Matrix

		Satisfaction with income level	Religious spirituality	Instrumental tendencies of religion	Religious identity
Satisfaction with income level	*r*	-			
Religious spirituality	*r*	-0.348	-		
Instrumental tendencies of religion	*r*	-0.406	0.539	-	
Religious identity	*r*	-0.383	0.568	0.579	-

**Table 10:** Model Coefficients - Irrational Religious Orientation

Predictor variable	ratio	standard error	*t*	*p*
intercept (the point at which a line crosses the x- or y-axis)	6.417	0.0903	71.1	< 0.001
Satisfaction with income level	-0.378	0.0161	-23.5	< 0.001

## Discussion

### Exploratory factor analysis

In the present study, through the application of exploratory factor analysis (EFA), we succeeded in revealing three main underlying dimensions of Unconventional Religious Orientations, providing insights into understanding this complex psychological construct. First, the first factor is closely related to religious spiritual dependence, encompassing an individual’s belief in temple wishing and the tendency to seek religious comfort under life stress. The second factor reflects religious instrumental tendencies, such as discussing religious experiences in social situations or spending money to seek religious solutions. The third factor, on the other hand, seems to be related to the identification of unique attributes of religion, including the consideration of religion in decision-making or the sense of belonging to a specific religious group. The findings of these three dimensions initially validate the related concepts proposed in Zagan’s theory, further capturing the multidimensionality and complexity of Unconventional Religious Orientations.

From a psychometric perspective, the scale was revised with the aim of enhancing its measurement validity and precision. In this process, we decided to remove the entry “T12. I would like to meet more monks and Taoist masters in real life so that my future will be secure”, a decision based on several key considerations. First, the factorial loading of this entry was below 0.5, suggesting that its contribution to the scale is relatively small and may not be sufficient to effectively represent the psychological construct to which it belongs [37]. In psychological scale construction, entries with high factor loadings are critical for accurately reflecting the psychological constructs studied. Therefore, removing low-loading entries helps to improve the overall validity and accuracy of the scale.

In addition, given the multidimensional nature of the scale as implied by the correlations between factors, removing entries with low factor loadings helped to maintain the focus and consistency of the scale, ensuring that it more accurately mapped the key dimensions of Unconventional Religious Orientations [31, 38, 39]. This revision strategy is intended to reduce measurement error or interference and allow the scale to be more focused on measuring the core aspects of Unconventional Religious Orientations. In summary, by removing specific low validity entries, we were able to enhance the accuracy and psychometric validity of the scale in measuring Unconventional Religious Orientations, providing a more reliable and valid measurement tool for future psychological research.

### Confirmatory factor analysis

This study applied the revised scale in a new sample to collect data for confirmatory factor analysis (CFA). This step was critical to validate the consistency and reliability of the scale across samples. By repeatedly applying the scale in different groups and collecting a large amount of data, we were able to more accurately assess the generalizability and measurement consistency of the scale.

The process of performing the CFA was central to this study The results of the CFA showed a good fit between the factor structure of the scale and the theoretical model. For example, the high factor loadings for the factors Religious Spiritual Dependence, Religious Instrumental Tendency, and Religious Uniqueness Identity confirmed the strong correlations between the scale entries and their respective factors. This not only demonstrates the validity of the scale in measuring the various dimensions of Unconventional Religious Orientations, but also emphasizes the strong alignment of the scale at the theoretical and empirical levels.

The meticulous validation of the scale by CFA not only confirmed the construct validity of the scale, but also highlighted the scientific rigor and empirical basis of the scale in measuring Unconventional Religious Orientations. The high factor loadings of the factors in this study, such as Religious Spiritual Dependence, Religious Instrumental Tendency, and Religious Uniqueness Identity, effectively captured the different dimensions of Unconventional Religious Orientations. These results not only reveal the multiple roles of religiosity in individuals’ coping with life stress, realizing personal goals, and constructing identity, but also provide a solid quantitative tool for future research on the psychology of religion in different cultural and social contexts. Overall, the scale validation work in this study enhances our understanding of the complexity of Unconventional Religious Orientations and paves the way for further theoretical development and practical application.

### The relationship between income level satisfaction and unconventional religious orientation

In this study, we focus on the link between income satisfaction and Unconventional Religious Orientations among Generation Z. The results point to a clear trend: as individuals become more satisfied with their income levels, their tendency to rely on religion for psychological comfort diminishes. The results point to a clear trend: as individuals become more satisfied with their income levels, their tendency to rely on religious beliefs for psychological comfort diminishes. This phenomenon is of particular interest in the field of psychology because it highlights the potential influence of economic satisfaction in shaping religious attitudes and behavior [40, 41].

In-depth analysis reveals that when Generation Z in Chinais faced with financial difficulties, they tend to turn to religion to satisfy their spiritual needs and seek a deeper meaning in their lives. This behavior reflects the complex interaction between material and spiritual needs in modern society. Particularly in an economically depressed environment, irrational religious behavior may become a psychological strategy for dealing with financial stress and uncertainty [42]. This strategy may partly explain why such religious tendencies rise under poorer economic conditions.

However, it is important to be wary of the potential risk that this Unconventional Religious Orientation may pose to young people in Generation Z. Some young people may seek religion as a psychological escape or seek non-traditional economic relief, such as seeking direct material benefits through religion. These behaviors may not only lead individuals to make poor economic decisions, but may also exacerbate reliance on irrational beliefs, thereby increasing individuals’ vulnerability in the face of economic hardship [43].

This study further suggests that socioeconomic status has a non-negligible impact on religious practices and belief formation. In situations of low economic satisfaction, religion may be seen as a channel of social support or as a way for individuals to affirm their personal values [44–46]. This phenomenon is particularly evident among Generation Z, who may view religion as a transaction rather than merely a spiritual quest.

To fully understand this complex phenomenon, future research should consider more possible mediating and moderating variables, such as educational background, cultural values, and social support systems. Meanwhile, enlarging the sample size and validating the applicability of the scale in different cultural contexts are also key tasks in further research. By doing so, we can gain a deeper understanding of the relationship between income satisfaction and religious mental states, and hopefully apply this knowledge in theory and practice to support Gen Z youth in making more informed choices, both financially and spiritually.

## Conclusion

The purpose of this study is to explore the relationship between Unconventional Religious Orientations and income level satisfaction in Generation Z. Using a self-constructed scale, we found that income level satisfaction showed a significant negative correlation with Unconventional Religious Orientation. This finding may reflect the increased reliance on irrational religion of Generation Z in Chinawhile failing to satisfy their income under the influence of their particular socio-economic background and values.

This study contributes significantly to both theoretical and empirical research. Theoretically, this study enriches the literature on the relationship between Generation Z’s economic satisfaction and religious behavior by providing new quantitative tools, as well as supporting related theories in economic psychology and psychology of religion. Empirically, through the application of a self-constructed scale, we not only verified the reliability and validity of the scale, but also realized an accurate measure of Generation Z’s Unconventional Religious Orientations.

Although the study provides valuable insights, there are limitations. The scale development process may be limited by sample selection and cultural context, and future research should consider a wider and more diverse sample, as well as validation of the scale’s applicability in different cultural contexts. In addition, further research on Unconventional Religious Orientations of Generation Z in Chinashould consider more dimensional economic variables, as well as other psychosocial factors that may affect religious tendencies.

In summary, this study explored the relationship between satisfaction with income level and Unconventional Religious Orientation of Generation Z in Chinathrough a carefully constructed scale, which provides a new perspective for understanding the religious and economic behaviors of this generation and points to a new direction for future research. These findings are not only valuable academically, but can also provide guidance for relevant policymaking and social interventions in practice.

## Data Availability

No datasets were generated or analysed during the current study.
